# Palliative Care Emergency: A Rare Etiology of Acute Pain

**DOI:** 10.7759/cureus.27924

**Published:** 2022-08-12

**Authors:** Reshmi Mathew, Amy Roemer, Eby Thekkedath, Ravindra P Maharaj

**Affiliations:** 1 Internal Medicine, University of Florida College of Medicine – Jacksonville, Jacksonville, USA; 2 Internal Medicine, Geriatrics, and Palliative Care, University of Florida College of Medicine – Jacksonville, Jacksonville, USA

**Keywords:** pet scans, cancer and non cancer pain, pneumothorax (ptx), palliative care, hospice and palliative care

## Abstract

Patients with serious or life-threatening illnesses are typically referred to palliative care to discuss goals of care, advance care planning, and to seek control of their cancer-related pain. Physicians who care for patients near the end of life quite often attribute worsening pain to advancing disease. We present a case of a patient with metastatic gallbladder adenocarcinoma who presented to a palliative care clinic with complaints of worsening chest and back pain, uncontrolled with her established opioid pain regimen. Findings on physical examination prompted the search for other etiologies of this patient’s worsening pain. An initial review of her recent investigations revealed a suspicious positron emission tomography (PET) scan obtained prior to her clinic appointment, which showed a large right-sided pneumothorax with tension physiology. The patient was urgently sent to the emergency room for emergent placement of a chest tube. This case attempts to bring awareness to the potential bias physicians may have regarding the pain experienced by patients with advanced disease and who are near the end of life. The performance of a thorough physical examination can be neglected in a developed, resource-rich country where imaging is easily accessible. Although the adoption of a stepwise ladder in pain management for patients at the end of life is frequently implemented, forgoing a thorough history and physical examination can have detrimental effects. Consideration of other etiologies of acute pain remains imperative when treating patients at the end of life.

## Introduction

Physicians encounter a wide array of symptoms in patients with cancer, including but not limited to pain, dyspnea, anxiety, depression, and fatigue [1]. The management of pain in patients at the end of life should include a thorough evaluation of the primary diagnosis and the extent of involvement of other organ systems [2]. A thorough evaluation should start with a comprehensive patient interview [2]. This should be followed by a thorough physical examination to assess for various factors that could be contributing to pain [2]. It is important for physicians to consider other etiologies for pain in cancer patients even at the end of life. It is imperative for physicians to remain knowledgeable about the serious and complex illnesses encountered in patients at the end of life and be familiar with how to manage palliative emergencies [1].

## Case presentation

A 53-year-old female with multiple hospital admissions related to abdominal pain presented with complaints of severe, sharp right-sided chest and abdominal pain with radiation to her back. Computed tomography (CT) of the abdomen showed an ill-defined heterogeneity and hypodensity in the gallbladder fossa with adjacent edema that was concerning for malignancy. Biopsy of the mass revealed gallbladder mucinous adenocarcinoma. Initial CT of the chest after the diagnosis of gallbladder adenocarcinoma showed no intrathoracic lymphadenopathy or lung metastases. A follow-up PET scan was ordered for staging purposes to be completed on an outpatient basis. Oncology planned to start the patient on a chemotherapy regimen once she was medically optimized to include gemcitabine and cisplatin every three weeks. Palliative care was also consulted for the management of her cancer-related pain. She was initially started on a pain regimen inpatient that included hydromorphone and morphine sulfate extended-release tablets for the treatment of severe cancer-related pain.

**Figure 1:**
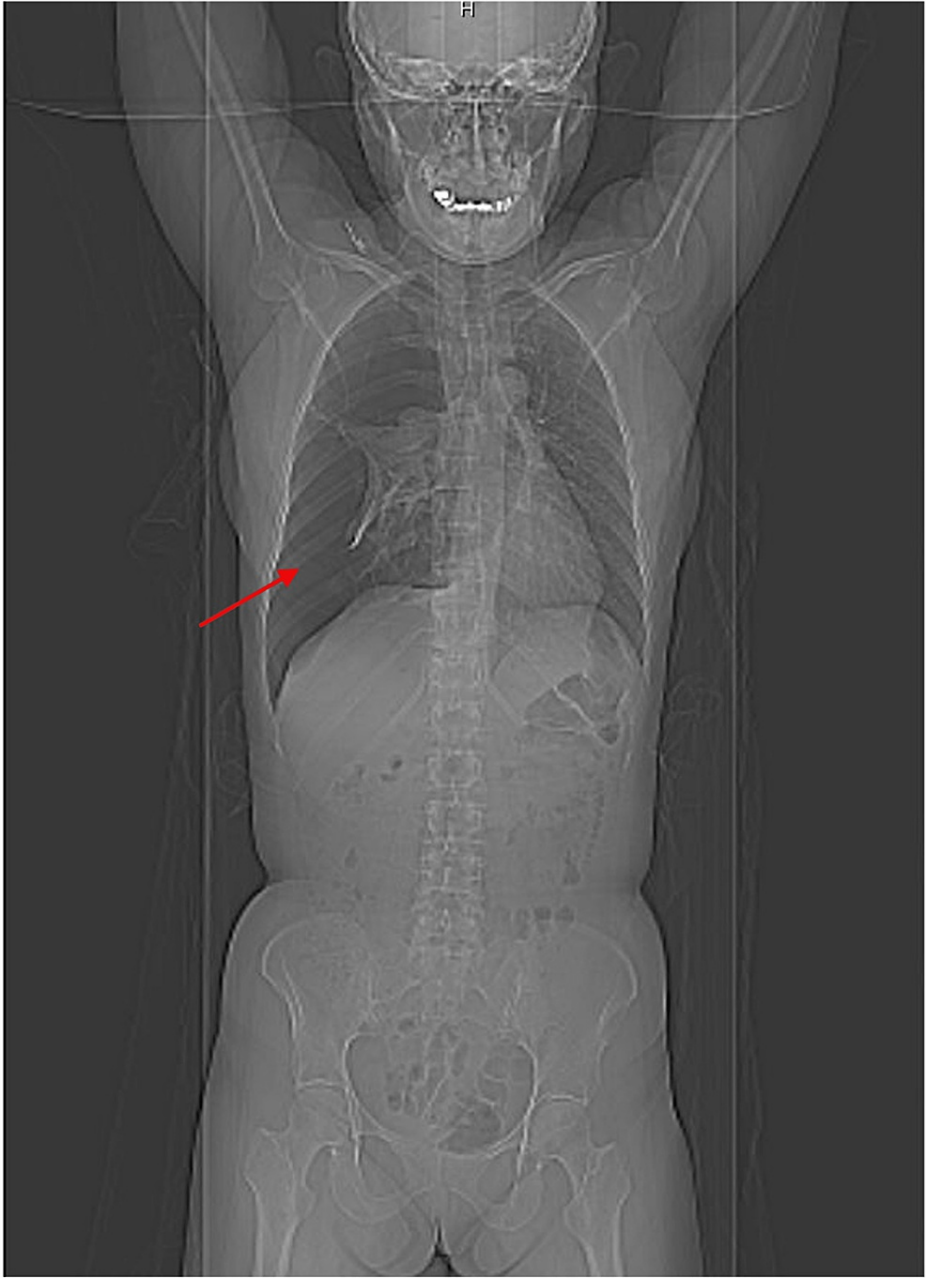
PET/CT showing hyperlucency of the right-sided lung (arrow) in coronal view.

The patient was followed up in an outpatient palliative care clinic with complaints of severe abdominal and back pain that was unrelieved by her established opioid regimen. Physical examination revealed a cachectic patient in moderate distress. The examination was remarkable for the complete absence of breath sounds on the right-sided lung fields. She was euvolemic on examination, with no evidence of jugular venous distension or pedal edema. The palliative care team started to further investigate the patient’s case with a chart review including prior imaging studies of her chest. Multiple imaging scans of the chest, including chest x-rays (CXR) and CT scans, had no acute abnormalities. However, the patient had a PET scan completed days prior to her current visit that had not yet been read by a radiologist. The most remarkable finding on the PET scan was the incidental finding of a large right-sided pneumothorax (Figure 1).

**Figure 2:**
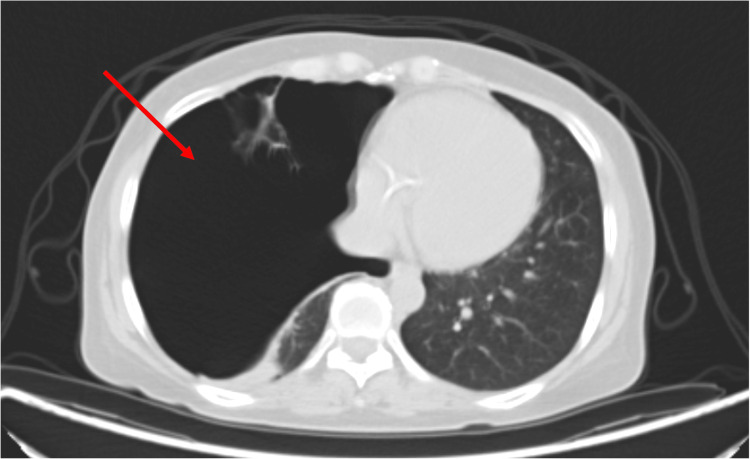
PET/CT scan showing a large right-sided pneumothorax (arrow) with near complete atelectasis of the remaining right hemithorax.

Closer evaluation of the chest portion of the PET scan is shown in Figure 2.

**Figure 3:**
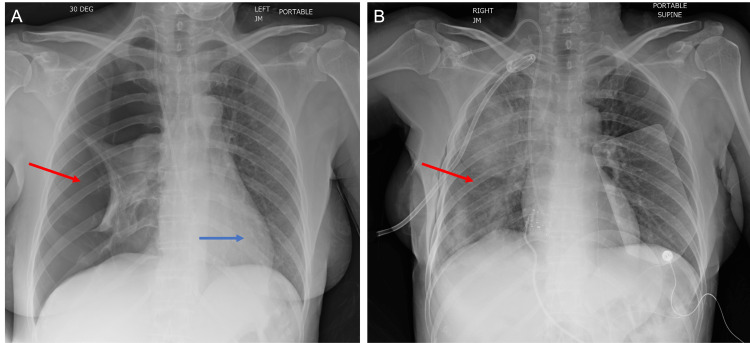
(A) CXR on admission again showing a large right-sided pneumothorax (red arrow) with an element of tension physiology demonstrated by leftward deviation of the heart (blue arrow) and mediastinal structures. (B) CXR showing reexpansion of the right-sided lung (arrow) after interval placement of a pigtail catheter.

The patient was urgently sent to the emergency department for further management. The patient developed acute hypoxic respiratory failure, requiring intubation for mechanical ventilation. She had an aspiration event prior to intubation and developed septic shock shortly thereafter, requiring pressors for vasoactive support. CXR on admission redemonstrated a large right-sided pneumothorax with tension physiology evidenced by leftward deviation of the heart and mediastinal structures (Figure 3A). The patient had placement of a right-sided chest tube. Repeat CXR showed the total resolution of the pneumothorax seen on initial imaging (Figure 3B).

The patient was extubated two days after admission. The chest tube was removed three days after admission. She continues to follow up with oncology and palliative care for ongoing care.

## Discussion

Our patient had multiple admissions with complaints of chest pain, abdominal pain, and back pain. After her diagnosis of metastatic gallbladder carcinoma, she was started on a pain regimen that included hydromorphone and morphine sulfate extended-release tablets. Due to multiple presentations secondary to worsening pain presumed secondary to cancer progression, there was consideration for alternative approaches to pain management, such as celiac plexus nerve block. Because the physicians in the palliative clinic prioritized comprehensive patient care in this case, it allowed for prompt recognition of an unexpected diagnosis that has the potential for serious morbidity and mortality if left untreated. This allowed the patient to receive prompt intervention and prevention of possible death.

The exact etiology of this patient’s pneumothorax remains unknown. However, the patient had a placement of a right-sided chemotherapy port three weeks prior to the diagnosis of pneumothorax on a PET scan. It is hypothesized that the patient developed a pneumothorax as a complication of chemotherapy port insertion. It is important to note, however, that CXRs completed after insertion of the chemotherapy port did not show evidence of pneumothorax. Pneumothoraces have also been shown to occur in the setting of malignancy. Clinicians should consider the possibility of lung metastases in a patient with the development of pneumothorax with known malignancy. However, our patient had no evidence of metastases to the lungs at the time of this evaluation.

The most common symptoms encountered in the palliative care setting include pain, dyspnea, anxiety, depression, and fatigue [1]. The treatment of both acute and chronic pain remains a cornerstone of palliative care [1]. The management of pain at the end of life can include pharmacologic and nonpharmacologic means of treatment. Pain medications, such as opiates, are widely used in this patient population. The World Health Organization formulated a cancer pain ladder to guide the selection of pharmacologic agents in pain management [3]. This approach encourages the initial use of non-opioids with incremental increases in doses of opioids and other agents to alleviate pain [2]. The nonpharmacologic approach to pain includes, but is not limited to, proper positioning and repositioning of the patient’s body to avoid pressure ulcers and dressing changes in areas of skin breakdown. It is also important to consider other conditions that can mimic pain at the end of life. Severe dehydration can lead to an altered mental status that can be mistaken for pain and discomfort [2]. Starting or increasing the dose of pain medications in these patients can lead to a worsening of mental status. Furthermore, long-term use of opiates can lead to opioid hyperalgesia leading to worsening pain [2].

Emergencies in palliative care can include hemorrhage, convulsions, fractures, spinal cord compression, and acute confusion [4]. Acute severe exacerbation of symptoms, such as the acute onset of severe pain or shortness of breath, is also considered a palliative care emergency because it has a significant impact on the quality of life for these patients [4,5]. It has been reported that 65-85% of cancer patients experience breakthrough pain, unlike the pain that is typically medically controlled [5]. It is generally accepted that breakthrough pain can be treated with fast and short-acting opioids [5]. However, had the management in this case been an escalation in pain medication, a significant diagnosis could have been missed. There should always remain a suspicion of other etiologies of pain in patients at the end of life.

Physicians who frequently care for patients with cancer and those near the end of life can develop a bias toward labeling all pain experienced by their patients as occurring secondary to the progression of their disease. However, it is important for physicians to remember that palliation should continue to encompass comprehensive medical care for patients and should not only be considered an alternative when traditional medical therapies have failed [1]. Comprehensive patient care includes a thorough history and physical examination, in addition to carefully considering alternative etiologies for acute or worsening pain that patients at the end of life present with.

## Conclusions

Patients at the end of life often seek palliative care to help alleviate the pain associated with their progressive condition. Physicians who frequently care for patients at the end of life may have premature closure with respect to pain in a known cancer patient. However, it is imperative for physicians who care for patients at the end of life to complete a thorough interview and physical examination to exclude other causes of acute and chronic pain. This case presents a near miss of a pneumothorax first diagnosed on a PET scan in the outpatient setting. The physicians in this palliative care clinic performed a comprehensive interview and physical examination. Physical examination findings raised suspicions about this patient's breakthrough pain; a recent PET scan revealed the incidental finding of a large pneumothorax, explaining this patient’s worsening pain and discomfort. This allowed us to redirect this patient to the emergency room to receive timely intervention and prevention of serious morbidity and mortality. Had the physicians in this clinic chosen to simply escalate this patient’s pain regimen, she could have experienced serious complications or premature death. This case highlights why it is important for physicians who treat patients at the end of life to consider other etiologies of pain to provide the best and most comprehensive care for these patients.
